# Chemical Composition, Antibacterial and Phytotoxic Activities of *Peganum harmala* Seed Essential Oils from Five Different Localities in Northern Africa

**DOI:** 10.3390/molecules21091235

**Published:** 2016-09-15

**Authors:** Ida Apostolico, Luigi Aliberti, Lucia Caputo, Vincenzo De Feo, Florinda Fratianni, Filomena Nazzaro, Lucèia Fàtima Souza, Maroua Khadhr

**Affiliations:** 1 Department of Pharmacy, University of Salerno, Via Giovanni Paolo II, 132, 84084 Fisciano (Salerno), Italy; i.apostolico1@studenti.unisa.it (I.A.); luigialiberti83@libero.it (L.A.); lcaputo@unisa.it (L.C.); luceia.souza@ufrgs.br (L.F.S.); 2 Istituto di Scienze dell’Alimentazione, Consiglio Nazionale delle Ricerche (ISA-CNR), via Roma 64, 83100 Avellino, Italy; florinda.fratianni@isa.cnr.it (F.F.); mena@isa.cnr.it (F.N.); 3 Post-doctoral by National Counsel of Technological and Scientific Development, (CNPq/Brazil), 70000-000 Brasília, Brazil; 4 Unité de Recherche de Biochimie des Lipides et Principes Actifs des Plantes, 2092 Faculté des Sciences de Tunis, Tunisa; miro0709@yahoo.fr

**Keywords:** *Peganum harmala*, essential oil, antibacterial activity, phytotoxic activity

## Abstract

*Peganum harmala* L., also known as Syrian rue or Pègano, is a herbaceous plant belonging to the Zygohpyllaceae family, and is widely used in traditional medicine. The chemical composition of essential oils of *P. harmala* seeds from five different regions of Northern Africa (Algeria, Egypt, Libya, Morocco and Tunisia) was studied by GC and GC-MS analyses. A total of 105 compounds were identified, the main components being oxygenated monoterpenes and oxygenated sesquiterpenes. Eugenol is the main component in all oils. The antimicrobial activity of the essential oils was assayed against some bacterial strains: *Staphylococcus aureus* (DSM 25693), *Bacillus cereus* (DSM 4313), *Bacillus cereus* (DSM4384), *Escherichia coli* (DMS 857) and *Pseudomonas aeruginosa* (ATCC 50071). All the oils showed different inhibitory activity. In the twentieth century this is an important result; we need possible new botanical drugs because the problem of resistance to antimicrobial drugs has become apparent. Moreover, the essential oils were evaluated for their possible in vitro phytotoxic activity against germination and initial radicle growth of *Raphanus sativus* L., *Lepidium sativum* L., and *Ruta graveolens* L. The results showed that both germination and radical elongation were sensitive to the oils.

## 1. Introduction

*Peganum harmala* L., also known as Syrian rue or Pègano, is a herbaceous plant belonging to the Zygohpyllaceae family. It is native to arid regions ranging from the eastern Mediterranean to northern India, Mongolia, and is present in South-East Europe, including southern Italy (Puglia and Sardinia) in arid steppes and sides of roads [1].

The seed oil is marketed in Egypt as an aphrodisiac. In ancient times, seeds and other parts of the plant were burned to produce a dense smoke during Zoroastrians rituals, and this use survives today. In Ladakh, India, the seeds of *P. harmala* are roasted and pulverized to obtain a fine powder, called *techepakchiatzen*, taken alone or smoked with tobacco to obtain narcotic effects [2]. In Iran, the smoke of its seeds is traditionally used as a disinfectant [3]. Different parts, including seeds, fruits, roots, are traditionally used as emmenagogues, anti-helmintics, soporifics, narcotics, aphrodisiacs, lactagogues, abortifacients and in treatment of fever, rheumatism and asthma as well as eye disorders [4,5]. Extracts of the plant are also used for recreation and as a stimulant of the central nervous system [6]. Moreover, antimicrobial, antifungal and anti-parasitic properties are attributed to *P. harmala* in India and North Africa [7].

The plant is rich in indole alkaloids in all its parts, and these are particularly abundant in the seeds, rather than in the other organs of the plant [8]. The seeds are also a rich source of β-carboline alkaloids [9,10]. The profile of alkaloids in seeds and roots is richer, followed by leaves and stems and their presence, as well as the presence of other secondary metabolites, could explain the plant´s toxic effect on animals [11].

Only a few studies are available in literature about the chemical composition of essential oils (EOs) from *P. harmala*. The composition of the essential oil from different plant parts has been reported, from plants grown in Egypt [12] and Morocco [13].

Extracts and essential oils of *P. harmala* have been reported for antimicrobial [14,15] and antitumoral activities [16]. The acute toxicity of the essential oil has been evaluated [12]. Extracts of *P. harmala* have been reported for insecticidal activities against *Tribolium castaneum* [17].

The alkaloids of the plant have been extensively studied and, in some cases, related mechanisms of action have been hypothesized. The hypothermic effect has been linked to serotonergic mechanisms [18]. Vasorelaxant activity of seed alkaloids has been reported in animal models [19,20,21]. It has been demonstrated that harmaline, harmine and harmalol decrease systemic blood pressure and total peripheral vascular resistance [22], and that α-carboline alkaloids possess anti-platelet activity [23]. Alkaloids from *P. harmala* also showed analgesic [24], anti-nociceptive [25], antibacterial and antifungal [26] activities. The activity of the plant alkaloids on CNS have been reported [10].

In this paper we studied the composition of the essential oils from *P. harmala* seeds from five countries in Northern Africa and their possible antimicrobial and phytotoxic activities. However, this study underlines that for production of botanical drugs it is necessary to uniform agriculture practices because finished products may be influenced by many intrinsic (genetic) or extrinsic (collection methods, cultivation, harvest) factors [27,28].

## 2. Results

### 2.1. Chemical Composition of Essential Oils

**Figure 1:**
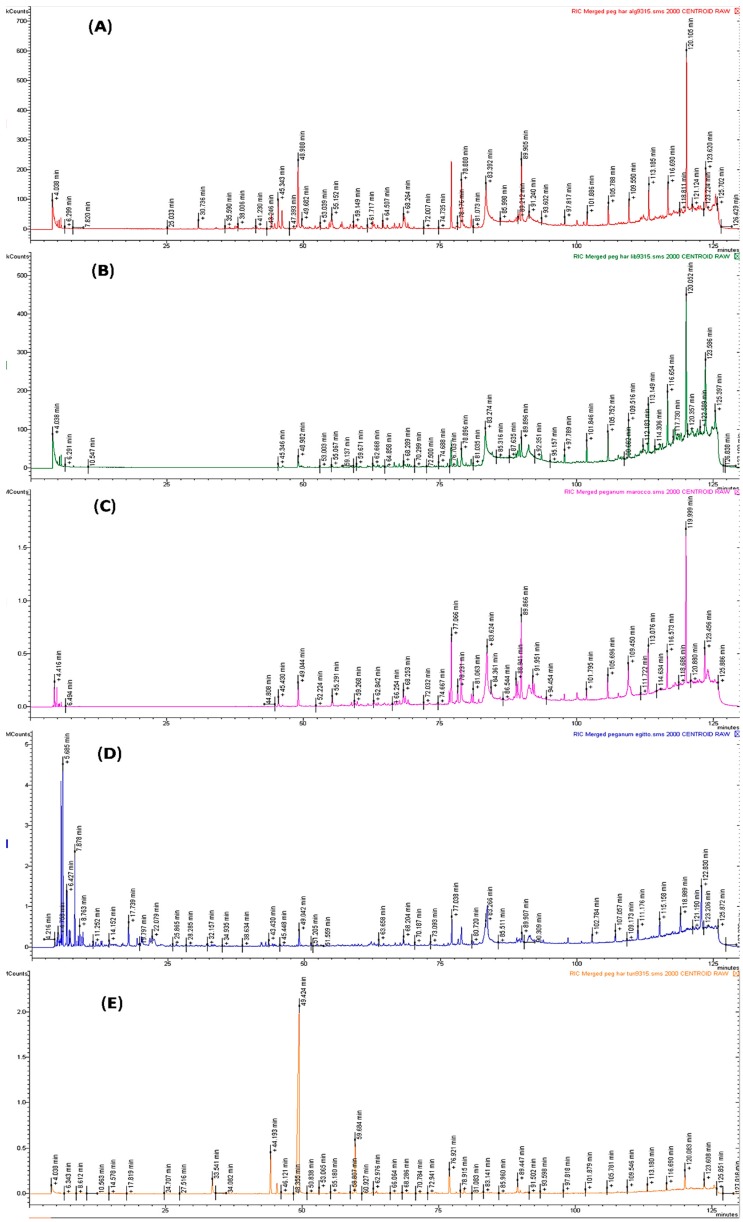
The Gas Chromatography (GC) profile of *P. harmala* essential oil from Algeria (**A**); Egypt (**B**); Libya (**C**); Morocco (**D**) and Tunisia (**E**).

Hydrodistillation yielded 0.005%, 0.003%, 0.002%, 0.001% and 0.01% of essential oil (on a dry mass basis) for *P. harmala* from Algeria (A), Egypt (E), Libya (L), Morocco (M) and Tunisia (T), respectively. Table 1 shows the chemical composition of the five *P. harmala* oils; compounds are listed according to their elution order on a HP-5MS column. The GC profile of five essential oils are present in Figure 1. Altogether, 105 compounds were identified, 45 for *P. harmala* from A, 38 for E, 20 for L, 38 for M and 37 for T, accounting for 89%, 93.3%, 95.4%, 75.72% and 95% of the total oil compositions.

### 2.2. Antimicrobial Activity

Using the agar diffusion test, we evaluated the potential antimicrobial activity of the EOs obtained from *P. harmala* grown in the five countries of the Mediterranean area. All samples were capable of inhibiting the growth of the bacteria used as tester strains (Table 2). *Escherichia coli* is more sensitive to all oils, especially at a concentration of 15 μg/mL, with an inhibition area of 10.0 mm using the Egyptian oil. This value was higher than that shown by the control tetracycline against the same microorganism (6.0 ± 0.5 mm). The essential oil from Egypt showed quite similar effects against *Bacillus cereus* DSM 4384 at the same concentration. However, *Staphylococcus aureus* is more sensitive to essential oil from Morocco. *Bacillus cereus* DSM 4313 was the least sensitive to lower concentrations, but still shows a zone of inhibition of 9 ± 0 mm when the tests were effectuate with the essential oil from Libya at 15 μg/mL.

### 2.3. Phytotoxic Activity

The five essential oils were evaluated for their activity against germination and radicle elongation of radish (*Raphanus sativus* L.)—a species frequently utilized in biological assays—of garden cress (*Lepidium sativum* L.), and rue (*Ruta graveolens* L.). The five oils seem to be effective against germination of all these species (Table 3). In particular, treatment of seeds with concentrations of 100 μg/mL of the oil from Algeria is the most active against germination of radish. The concentration of 25 μg/mL of essential oils from Libya and concentrations of 50 μg/mL and of 25 μg/mL of essential oil from Tunisia inhibited significantly the germination of garden cress.

The essential oils from Algeria, Egypt and Libya, at all doses tested, significantly inhibited the radicle elongation of *R. graveolens*. However, concentrations of 25 μg/mL and 12.5 μg/mL of the oil from Tunisia are active against the germination of seeds oh the same species. The five essential oils affected significantly, at all doses tested, the radicle elongation in *R. graveolens* (Table 4).

## 3. Discussion

Oxygenated monoterpenes are highly predominant in *P. harmala* essential oils from A, L, M and T. Non-terpenic compounds are the main constituents of essential oils. In all oils, eugenol (13.2%–69.2%) was the most abundant component. Another component present in substantial quantity is thymol (6.9% for A, 5% for L, 5% for M and 1.8% in T). However this component is not found in essential oil from Egypt. Our results corroborate previous studies reporting the presence of eugenol but does not report the presence of thymol in the *P. harmala* essential oil from Egypt [12]. Tahrouch et al. [13] showed the presence of thymol in an essential oil from Morocco, but not of eugenol.

Chemical profiling is only basic, however DNA profiling will provide an additional safeguards on quality authentication. In fact, if the active components of a medicinal plant or essential oil become known and used as active treatment agent, uniformity will become mandatory and Good Agricultural Practice (GAP) should be practiced and enforced [27].

The composition of the essential oil of *P. harmala* in different countries is quite varied, suggesting that different factors can affect the oil composition. Optimization, standardization, and full control of growing conditions can guarantee quality-controlled production of plant-derived compounds [28]. In fact, the development stage is one of the determining factors of the yield and composition of essential oil [29]. In many cases an increase of the yield of oil from the bud stage to mature flower can change the chemical composition of the oil, and in some cases in the early stages this can vary more by than 10%. In *Ocimum* spp., the relative concentration of eugenol decreased with the development of the leaves, probably depending on the use of these compounds in the synthesis of lignin [30]. According to Manez et al. the alteration of the composition of the essential oil with maturation is closely related to the biosynthetic pathways leading to greater cyclization and dehydration of oil components [31].

Generally, the antimicrobial activity of *P. harmala* is attributed to specific components present in the hydroalcoholic or hexane portion of plant [32,33,34]. In our findings, few studies ascertained the antimicrobial potentialities of *P. harmala* essential oil [35]. Selim et al. [12] demonstrated the antimicrobial activity of *P. harmala* oil against *B. cereus*, *S. aureus* and *E. coli*, even if they used a higher concentration. We did not find an evident difference in sensitivity between the Gram+ and Gram− strains used in the experiments.

Taking into account the chemical composition of the five EOs, the difference in bioactivity could be attributable to the presence of nerol acetate [36], or to the different content of oxygenated sesquiterpene. The presence of eugenol, active on bacterial membranes is also of interest.

Eugenol, as isoeugenol, vanillin, and cinnamaldehyde are safrole phenylpropenes with antimicrobial activity [37], with isoeugenol more active than eugenol. Data show that the effectiveness of the oils is not strictly correlated to the percentage of eugenol, in fact the oil from M with a lower percentage of eugenol (13.2%) showed similar or higher antimicrobial activity than the others where eugenol was present at 17.8% or 69.2%. This shows that often the biological properties cannot be attributed to a single compound but to the synergy between the different components. In our study we evaluated the possibility of using the essential oil and not a singular component (eugenol) whose activity was already shown in previous studies. In our view, the use of *P. harmala* essential oil could impulse and improve the economy of these areas of the Mediterranean.

In all cases, the antimicrobial activity exhibited by the *P. harmala* oil against both Gram-positive (*B. cereus* and *S. aureus*) and Gram-negative (*E. coli* and *P. aeruginosa*) bacteria may indicate the presence of a broad spectrum of compounds with antibiotic activity. *E. coli* and *S. aureus* resulted generally more sensitive to the action of the 5 μg of the five *P. harmala* oils, in comparison to *Pseudomonas aeruginosa*, at their lowest concentration. We cannot attribute the different antimicrobial activity to a specific compound, present or absent in significant way in the two EOs from Libya and Tunisia, nor could this explain the slightly different behavior exhibited by the *B. cereus* 4384, which showed more sensitivity with low concentrations (5 and 10 μg) of EO used in the experiments. The antimicrobial compounds are commonly found in the essential oil fractions and many have a wide spectrum of antimicrobial activity [38]. Probably the method of extraction of essential oils influences their chemical composition and consequently their biological activities, in fact Shaverdi et al. have not observed antimicrobial activity of their *n*-exane smoke condensed extract derived from *P. harmala* seeds [3]. The main compounds of their smoke preparation are totally different from essential oil ones except for two compounds: hexadecane and heptadecane, this could explain the different antimicrobial activity.

The difference in phytotoxic activity of the oils could be attributed to their chemical composition. Oxygenated monoterpenes are predominant in essential oils from Algeria, Libya, Morocco and Tunisia. Our results agree with previous studies showing that monoterpenes and essential oils possess potent herbicidal effects on weed germination and seedling growth of various plant species [39,40,41,42,43]. The main monoterpene of five essential oils studied is eugenol. Vaid et al. [44] reported that eugenol suppresses some weedy species. Moreover, eugenol has been reported to damage plant tissue by damaging its cellular membranes [45,46].

## 4. Materials and Methods

### 4.1. Plant Material and Essential Oil Extraction

The seeds of *P. harmala* were collected from Tunisia (region of Kasserine-Bouzguem), Algeria (South of Algeria-Bousaada), Libya (region of El Hisha-South Derna), Morocco (Marrakech-Haouz) and Egypt (region of Marsa-Mattrouh). The main characteristics are reported in Table 5. The plant material was authenticated by Ms. Maroua Khadhr. The seeds were collected from individuals of the entire population to get an adequate representation of genetic diversity. Inflorescences were air-dried; seeds were separated from spikes and cleaned before surface. The seeds were dried at room temperature in the shade and weighed each day until the difference between two successive weights was less than 5%.

One hundred grams of dried seeds of each sample were ground in a Waring blender and then, subjected to hydrodistillation for 3 h according to the standard procedure described in the European Pharmacopoeia [47]. The oils were solubilized in *n*-hexane, filtered over anhydrous sodium sulphate and stored under N_2_ at +4 °C in the dark, until tested and analyzed.

### 4.2. GC-FID Analysis

Analytical gas chromatography was carried out on a Perkin-Elmer Sigma-115 gas-chromatograph (Pelkin-Elmer, Waltham, MA, USA) equipped with a FID and a data handling processor. The separation was achieved using a HP-5 MS fused-silica capillary column (30 m × 0.25 mm i.d., 0.25 μm film thickness). Column temperature: 40 °C, with 5 min initial hold, and then to 270 °C at 2 °C/min, 270 °C (20 min); injection mode splitless (1 μL of a 1:1000 *n*-hexane solution). Injector and detector temperatures were 250 °C and 290 °C, respectively. Analysis was also run by using a fused silica HP Innowax polyethylene glycol capillary column (50 m × 0.20 mm i.d., 0.25 μm film thickness). In both cases, helium was used as carrier gas (1.0 mL/min).

### 4.3. GC/MS Analysis

Analysis was performed on an Agilent 6850 Ser. II apparatus (Agilent, Roma, Italy), fitted with a fused silica DB-5 capillary column (30 m × 0.25 mm i.d., 0.33 μm film thickness), coupled to an Agilent Mass Selective Detector MSD 5973 (Agilent); ionization energy voltage 70 eV; electron multiplier voltage energy 2000 V. Mass spectra were scanned in the range 40–500 amu, scan time 5 scans/s. Gas chromatographic conditions were as reported in the previous paragraph; transfer line temperature, 295 °C.

### 4.4. Identification of the Essential Oil Components

Most constituents were identified using gas chromatography by comparison of their Kovats retention indices (R_i_) (determined relative to the *t*_R_ of *n*-alkanes (C_10_–C_35_)), with either those of the literature [48,49,50,51] and mass spectra on both columns or those of authentic compounds available in our laboratories by means of NIST 02 and Wiley 275 libraries [52]. The components’ relative concentrations were obtained by peak area normalization. No response factors were calculated.

### 4.5. Antibacterial Activity

The antibacterial activity was evaluated in vitro, by means of the agar diffusion test on the plate. The activity of the essential oils was tested on five species of bacteria; *S. aureus* (DMS 25693), *B. cereus* (DSM 4313) and *B. cereus* (DSM 4384), representative of the Gram-positives; and *E. coli* (DMS 857) and *P. aeruginosa* (ATCC 50071) for Gram-negatives.

Microbial strains were previously grown in Nutrient Broth (Sigma, Milano, Italy), at 37 °C for 18 h. The microbial suspensions (1 × 10^7^ cfu/mL) were uniformly distributed on Nutrient agar plates in sterile conditions. Different amounts of essential oils were spotted on the inoculated plates: 5 μg/mL, 10 μg/mL and 15 μg/mL. After 10 min, under sterile conditions, plates were then incubated at 37 °C for 24 h.

The antimicrobial activity performed with essential oils was evidenced by measuring the diameter (in mm) of the zone of inhibition. The diameter of the clear zone shown on plates was accurately measured using the “Extra steel caliper mod 0289”, mm/inch reading scale, precision 0.05 mm (Mario De Maio, Milan, Italy). Dimethylsulfoxide (DMSO) was the negative control; on the other hand, tetracycline (7 μg) and gentamycin (8 μg) were used as positive standards. The value of MIC was evaluated as the lowest concentration of the sample that did not allow any visible growth of the microorganisms after incubation. Each oil was tested in triplicate.

### 4.6. Phytotoxic Activity

The phytotoxic activity was evaluated on germination and root elongation of three different plant species: *R. sativus* cv ‘Saxa’ (radish), *L. sativum* (garden cress) and *R. graveolens* (rue). These seeds are usually used in assays of phytotoxicity because they easily germinable and well known from the histological point of view. The seeds of *R. sativus* and *R. graveolens* were purchased from Blumen group srl (Emilia Romagna), the seeds of garden cress from Euroselect (Bari). The seeds were surface sterilized in 95% ethanol for 15 s and sown in petri dishes (Ø = 90 mm), containing five layers of Whatman filter paper, impregnated with distilled water (7 mL, control) or the tested solution of the essential oil (7 mL), at the different doses. The germination conditions were 20 ± 1 °C, with natural photoperiod. The essential oils, in water–acetone mixture (99.5:0.5), were assayed at the doses of 100, 50, 25 and 12.5 μg/mL Controls performed with water-acetone mixture alone showed no appreciable differences in comparison to controls in water alone. Seed germination was observed directly in petri dishes every 24 h. A seed was considered germinated when the protrusion of the root became evident [53]. After 120 h (on the fifth day), the effects on radicle elongation were measured in cm. Each determination was repeated three times, using petri dishes containing 10 seeds each. Data are expressed as the mean ± SD for both germination and radicle elongation.

### 4.7. Statistical Analysis

All experiments were carried out in triplicate. Data of each experiment were statistically analyzed using GraphPad Prism 6.0 software (GraphPad Software Inc., San Diego, CA, USA), followed by comparison of means (one-way ANOVA) using Dunnett’s multiple comparisons test, at the significance level of *p* < 0.05.

## 5. Conclusions

We characterized the chemical composition of the essential oils of *P.harmala* grown in different Mediterranean countries. The composition of the essential oil of *P. harmala* in five countries is quite varied; in fact, the relative percentage of oxygenated monoterpenes, sesquiterpenes, and oxygenated sesquiterpenes are different. This could indicate a strong influence of the external environment on the metabolic pathway of the same plant. The studied samples showed antimicrobial activity against both Gram-positive and Gram-negative microorganisms, although it should be impossible to attribute such activity to a specific compound or group of biomolecules. However, the antimicrobial activity registered confirms some of the traditional uses of the plant. The essential oils studied showed different phytotoxicity, namely in the inhibition of radical growth of the three test species studied.

## Figures and Tables

**Table 1:** Essential oil (EO) compositions (%) of *Peganum harmala* from Algeria (**A**), Egypt (**E**), Libya (**L**), Morocco (**M**) and Tunisia (**T**).

N	RT ^a^	KI ^b^	Compounds	A	E	L	M	T	^c^
1	24.71	1009	*p*-Cymene	-	-	-	-	0.1	1, 2, 3
2	25.03	1016	Limonene	0.3	-	-	-	-	1, 2, 3
3	25.39	1018	2-Acetyl-Thiazole	-	1.3	-	-	-	1, 2
4	25.87	1024	Santolina alcohol	-	2.0	-	-	-	1, 2
5	26.49	1032	*cis*-Dihydro-rose oxide	-	1.8	-	-	-	1, 2
6	27.38	1044	1-Octen-ol	-	0.7	-	-	-	1, 2
7	30.08	1081	*trans*-Dihydro-Rose oxide	-	0.4	-	-	-	1, 2
8	30.73	1089	*n*-Octanol	-	1.1	-	-	-	1, 2
9	30.74	1090	Linalool	1.7	-	-	-	-	1, 2, 3
10	32.14	1109	*trans*-Thujone	-	-	-	-	0.1	1, 2, 3
11	32.16	1101	Methyl butanoate,3-methyl-3-butenyl	-	2.9	-	-	-	1, 2
12	32.94	1114	3-Decanone	-	1.1	-	-	-	1, 2
13	33.21	1118	Camphor	-	2.7	-	-	-	1, 2, 3
14	33.61	1127	Benzene acetonitrile	-	1.3	-	-	2.6	1, 2
15	35.59	1154	Isoborneol	0.9	-	-	-	-	1, 2
16	36.45	1166	Terpinen-4-ol	0.4	-	-	-	-	1, 2, 3
17	36.67	1169	Naphthalene	0.2	-	-	-	-	1, 2
18	37.45	1179	α-Terpineol	0.7	-	-	-	-	1, 2, 3
19	37.56	1180	1-Dodecene	-	0.7	-	-	-	1, 2
20	37.77	1184	(*E*)-Isocitral	0.7	-	-	-	-	1, 2
21	38.02	1187	Methyl chavicol	1.1	-	-	-	0.1	1, 2
22	40.72	1225	Isoquinoline	0.5	-	-	-	-	1, 2
23	41.23	1232	Carvone	0.7	-	-	-	-	1, 2
24	41.51	1236	Pulegone	0.5	-	-	-	-	1, 2, 3
25	42.20	1246	Cumin aldehyde	-	-	-	-	0.1	1, 2
26	44.19	1274	(*E*)-Anethol	3.7	-	-	-	6.9	1, 2
27	44.81	1283	α-Terpinen-7-al	-	2.2	-	1.0	0.1	1, 2
28	45.36	1291	Thymol	7.0	-	5.1	5.0	1.8	1, 2, 3
29	46.14	1301	Dihydro carveol acetate	6.2	1.0	3.0	0.7	0.3	1, 2
30	45.71	1297	Terpinyl acetate	-	0.9	-	-	-	1, 2, 3
31	48.70	1341	4-Methoxyacetophenone	1.0	-	-	-	-	1, 2
32	48.99	1346	Eugenol	17.5	17.2	17.8	13.2	69.2	1, 2, 3
33	49.68	1356	Cicloysosativene	2.3	-	-	-	-	1, 2
34	49.86	1359	α-Ylangene	1.1	-	-	-	-	1, 2
35	50.30	1366	Decanoic acid	-	1.0	-	-	-	1, 2
36	50.84	1374	Thujico acid	-	-	-	-	Tr	1, 2
37	51.18	1380	β-Cubebene	-	-	-	0.4	-	1, 2
38	51.21	1380	*n*-Undecanol	-	2.3	-	-	-	1, 2
39	51.34	1381	6,8-Nonadien-2-one,6-methyl-5-(1-methyletildene)	0.3	-	-	-	-	1, 2
40	52.13	1393	*iso*-Italicene	1.1	-	-	-	-	1, 2
41	52.16	1394	β-Longipinene	0.5	-	-	-	0.1	1, 2
42	52.17	1394	Methyleugenol	-	0.3	-	-	-	1, 2, 3
43	52.22	1395	β-Ionol	-	-	-	0.7	-	1, 2
44	53.07	1408	(*Z*)-Caryophyllene	2.3	-	0.3	-	0.8	1, 2
45	54.14	1425	(*E*)-α-Ionone	0.6	-	-	0.2	-	1, 2
46	55.07	1440	Nerol acetate	-	-	3.7	-	-	1, 2
47	55.11	1441	α-Isomethyl-(*E*)-ionol	7	-	-	-	-	1, 2
48	55.18	1442	Aromadendrene	-	-	-	-	0.3	1, 2, 3
49	55.29	1444	Bakerol	-	-	-	7.5	-	1, 2
50	56.55	1465	*trans*-Cadina-16,4-diene	-	-	-	0.3	-	1, 2
51	56.71	1467	9-*epi*-(*E*)-Caryophyllene	-	-	-	-	0.2	1, 2
52	56.79	1468	α-Acoradiene	0.5	-	-	-	-	1, 2
53	56.93	1471	γ-Gurjunene	-	-	-	0.6	-	1, 2
54	57.01	1472	γ-Muurolene	0.3	-	-	-	-	1, 2
55	57.21	1475	(*E*)-β-Ionone	-	-	-	0.6	-	1, 2
56	57.50	1480	(*E*)-Methyl isoeugenol	-	0.6	-	-	0.2	1, 2
57	57.52	1480	10,11-Epoxy-Calamenene	-	-	-	0.3	-	1, 2
58	58.09	1489	α-Zingiberene	0.3	-	-	-	-	1, 2
59	58.53	1496	γ-Amorphene	1.3	-	-	-	0.1	1, 2
60	58.77	1501	Methyl *p*-*tert*-buthylphenil acetate	0.3	0.8	-	2.0	0.2	1, 2
61	59.15	1507	10-Undecenol acetate	2.0	-	-	3.6	0.1	1, 2
62	59.26	1509	β-Curcumene	-	1.9	-	-	-	1, 2
63	59.50	1512	7-*epi*-α-Selinene	1.1	-	-	-	-	1, 2
64	59.69	1512	2*E*,4*E*-Dodecandienal	1.2	2.1	2.0	-	-	1, 2
65	59.56	1514	α-Thujaplicinol	-	-	-	4.8	-	1, 2
66	59.68	1516	Eugenol acetate	-	-	-	-	9.0	1, 2
67	60.24	1526	α-Bulnesene	-	-	-	0.4	-	1, 2
68	60.48	1529	(*Z*)-Nerolidol	0.5	1.6	2.5	1.3	0.1	1, 2
69	60.62	1531	α-Calacorene	0.6	-	-	0.6	-	1, 2
70	61.75	1551	Germacrene B	-	-	2.7	-	0.1	1, 2
71	61.79	1552	β-Calacorene	-	-	-	1.8	-	1, 2
72	62.30	1560	Dodecanoic acid	-	5.9	-	-	-	1, 2
73	62.71	1567	Spathulenol	2.0	2.3	4.2	4.0	0.2	1, 2
74	62.91	1571	Caryophyllene oxide	1.7	-	3.8	1.7	0.8	1, 2, 3
75	63.36	1579	1-Esadecene	-	1.6	-	0.9	0.2	1, 2
76	63.81	1586	Ledol	-	-	-	0.4	-	1, 2
77	63.86	1586	β-Oplopenone	-	-	-	-	0.1	1, 2
78	64.50	1597	*n*-Exadecane	2.6	-	2.8	2.9	0.1	1, 2
79	65.50	1614	Isolongifolan-7-α-ol	-	-	3.8	2.1	0.1	1, 2
80	65.57	1617	Cubenol	-	2.0	-	-	Tr	1, 2
81	66.06	1626	Caryophylla-4(12),8(13)-dien-5α-ol	-	-	-	-	0.1	1, 2
82	66.25	1629	*epi*-α-Cadinol	5.3	-	-	0.9	-	1, 2
83	66.62	1636	β-Acorenolo	1.5	2.9	7.4	2.3	0.1	1, 2
84	67.02	1643	*epi*-α-Muurololo	-	-	-	-	0.1	1, 2
85	67.03	1643	*cis*-Guai-3,9-dien-11-ol	0.7	-	-	-	-	1, 2
86	67.08	1645	*allo*-Aromadendrene epoxide	-	-	-	1.5	-	1, 2
87	67.53	1652	Vulgarone B	1.4	-	-	3.7	-	1, 2
88	67.55	1653	Cedr-8,15-en-10-olo	-	4.1	6.0	-	0.1	1, 2
89	67.93	1660	α-Eudesmol	-	1.6	-	-	-	1, 2
90	68.07	1662	14-Hydroxy-(*Z*)-Caryophyellene	-	-	2.1	-	0.2	1, 2
91	68.29	1666	*n*-Tetradecanol	4.8	12.3	11.3	11.1	0.3	1, 2
92	68.52	1670	Elemol acetate	0.7	-	-	-	-	1, 2
93	68.62	1671	*epi*-α-Bisabololo	-	3.0	6.6	-	-	1, 2
94	69.10	1681	Ciperotundone	1.6	3.0	4.6	4.1	0.1	1, 2
95	69.41	1686	Eptadecane	-	1.2	3.8	1.2	0.1	1, 2
96	69.74	1692	Calamenen-10-one	-	0.5	1.9	-	-	1, 2
97	70.19	1701	Longifolol	-	2.7	-	-	-	1, 2
98	70.26	1701	Farnesol	-	-	-	0.4	-	1, 2, 3
99	70.20	1701	3-Otadecine	0.3	-	-	-	-	1, 2
100	70.32	1704	Farnesale	-	1.0	-	-	-	1, 2
101	70.36	1704	Santalol	-	-	-	1.0	-	1, 2
102	71.45	1724	(*E*)-Nerolidil acetate	-	-	-	0.8	-	1, 2
103	72.28	1740	Amorpha-4,9-diene	-	0.3	-	1.6	-	1, 2
104	73.00	1753	Lanceol	-	-	-	1.5	-	1, 2
105	73.25	1758	14-Oxy-α-Muurolene	-	-	-	0.3	-	1, 2
Total %				89.0	92.3	95.4	75.7	95.0	
Monoterpenes				0.3	-	-	-	0.1	
Oxygenated monoterpenes				41.1	28.2	29.6	19.9	78.3	
Sesquiterpenes				11.4	2.2	3.0	4.1	1.6	
Oxygenated sesquiterpenes				15.4	25.6	42.9	40.5	11.4	
Non terpenes				20.8	36.3	19.9	11.2	3.6	

^a^ Retention time; ^b^ Kovats retention index determined relative to the *t*_R_ of a series of *n*-alkanes (C10–C35) on HP-5MS column; ^c^ 1 = Kovats retention index, 2 = mass spectrum, 3 = co-injection with authentic compound; Tr = trace (<0.1%).

**Table 2:** Antibacterial activity of essential oil of *P. harmala* from Algeria, Egypt, Libya, Morocco and Tunisia and of the reference compounds gentamicin and tetracycline. Results are expressed as the mean of three experiments ± standard deviation.

	*Bacillus Cereus* 4313	*Bacillus Cereus* 4384	*Escherichia Coli*	*Pseudomonas Aeruginosa*	*Staphylococcus Aureus*
Algeria					
5 μg/mL	n.a	2.7 ± 0.6 ****^,^****	7.0 ± 2 ****	4.7 ± 0.6 ****^,^****	4.7 ± 0.6
10 μg/mL	n.a	5.0 ± 0 ****^,^****	8.3 ± 1.2 ****^,^*	7.2 ± 2.0 ****^,^****	7.7 ± 1.2
15 μg/mL	n.a	7.0 ± 0 ****^,^****	9.3 ± 0.6 ****^,^***	8.0 ± 2.0 ****^,^**	10.0 ± 0 ****^,^****
Egypt					
5 μg/mL	n.a	3.3 ± 0.6 ****^,^****	5.7 ± 0.6 ****	n.a	5.3 ± 1.2
10 μg/mL	n.a	6.3 ± 0.6 ****^,^****	7.0 ± 0 ****	8.3 ± 0.6 ****^,^*	9.3 ± 1.0 ***^,^***
15 μg/mL	8.7 ± 1.2 ****^,^**	10.0 ± 0 ****	10.0 ± 0 ****^,^****	8.3 ± 0.6 ****^,^*	9.3 ± 1.2 ***^,^***
Libya					
5 μg/mL	4.3 ± 0.6 ****^,^****	5.0 ± 0 ****^,^****	5.0 ± 0 ****	5.0 ± 0 ****^,^****	5.0 ± 0
10 μg/mL	5.3 ± 0.6 ****^,^****	8.0 ± 0 ****^,^****	8.7 ± 0.6 ****^,^**	5.7 ± 0.6 ****^,^****	9.0 ± 2 **^,^**
15 μg/mL	9 ± 0 ****^,^*	9.3 ± 1.2 ****^,^*	8.7 ± 0.6 ****^,^**	8.7 ± 0.6 ****	9.3 ± 0.6 ***^,^***
Morocco					
5 μg/mL	n.a	5.3 ± 0.6****^,^****	6.0 ± 2.0 ****	4.3 ± 0.6 ****^,^****	5.0 ± 0
10 μg/mL	5.7 ± 1.2 ****^,^****	8.0 ± 0 ****^,^****	7.7 ± 0.6 ****	7.0 ± 0 ****^,^****	8.3 ± 0.6 *^,^*
15 μg/mL	7 ± 0 ****^,^****	9.0 ± 0 ****^,^**	9.0 ± 0 ****^,^***	7.3 ± 0.6 ****^,^***	10.7 ± 0.6 ****^,^****
Tunisia					
5 μg/mL	4.3 ± 0.6 ****^,^****	5.0 ± 0 ****^,^****	4.7 ± 0.6 ****	n.a	4.7 ± 0.6
10 μg/mL	5.7 ± 0.6 ****^,^****	7.0 ± 0 ****^,^****	6.3 ± 0.6 ****	6.7 ± 0.6 ****^,^****	7.0 ± 0
15 μg/mL	8.7 ± 0.6 ****^,^**	8.6 ± 0.6 ****^,^**	8.3 ± 0.6 ****^,^*	7.3 ± 0.6 ****^,^***	8.3 ± 0.6 *^,^*
		Control			
Gentamicin	17.7 ± 1.1	17.5 ± 0.5	16.5 ± 1.5	15.4 ± 1.8	6.1 ± 1.2
Tetracycline	10.5 ± 0.5	10.5 ± 0.5	6.0 ± 0.5	10.5 ± 0.5	6.0 ± 0.5

Dunnett’s test vs. control (gentamicin 7 μg, tetracycline 7 μg): **** *p* < 0.0001; *** *p* < 0.001; ** *p* < 0.01; * *p* < 0.05; n.a., not active.

**Table 3:** Phytotoxic activity of the essential oils of *P. harmala* from Algeria, Egypt, Libya, Morocco and Tunisia against germination of *Raphanus sativus* L., *Lepidium sativum* L., and *Ruta graveolens* L., 120 h after sowing. Results are expressed as the mean of three experiments ± standard deviation.

Germination					
*R. sativus*	Algeria	Egypt	Libya	Morocco	Tunisia
Control	6.6 ± 0.6	6.6 ± 0.6	6.6 ± 0.6	6.6 ± 0.6	6.6 ± 0.6
100 μg/mL	2.0 ± 1.0 **	4.0 ± 0	4.7 ± 1.5	5.0 ± 1.7	4.0 ± 1.7
50 μg/mL	4.0 ± 1.0 *	5.7 ± 1.5	4.7 ± 0.6	5.0 ± 2.0	4.0 ± 1.0
25 μg/mL	4.0 ± 1.0 *	4.7 ± 1.5	4.3 ± 0.6	5.0 ± 1.0	5.3 ± 1.2
12.5 μg/mL	3.3 ± 1.5 *	6.0 ± 1.7	4.3 ± 2.1	4.0 ± 1.0	5.3 ± 1.5
*L. sativum*					
Control	2.2 ± 1.3	2.2 ± 1.3	2.2 ± 1.3	NT	2.2 ± 1.3
100 μg/mL	2.3 ± 2.3	2.0 ± 1.7	2.3 ± 1.5	NT	1.7 ± 0.6
50 μg/mL	1.5 ± 1.0	2.7 ± 1.2	1.0 ± 1.0	NT	1.0 ± 1.0 *
25 μg/mL	1.0 ± 0	2.3 ± 0.6	0.3 ± 0.6 *	NT	1.0 ± 0 *
12.5 μg/mL	2.0 ± 0	3.0 ± 1.0	2.0 ± 1.0	NT	1.7 ± 1.2
*R. graveolens*					
Control	7.6 ± 1.5	7.6 ± 1.5	7.6 ± 1.5	NT	7.6 ± 1.5
100 μg/mL	2.0 ± 1.0 **	2.7 ± 0.6 ***	3.3 ± 1.2 **	NT	5.3 ± 1.5
50 μg/mL	2.7 ± 1.2 **	2.3 ± 1.2 ***	2.7 ± 0.6 **	NT	5.0 ± 2.0
25 μg/mL	2.7 ± 0.6 **	0	3.3 ± 1.2 **	NT	4.0 ± 1.0 *
12.5 μg/mL	2.3 ± 2.1 **	0	3.7 ± 1.5 **	NT	3.7 ± 1.5 *

Note: *** *p* < 0.001; ** *p* < 0.01; * *p* < 0.05 vs. control; NT (not tested).

**Table 4:** Phytotoxic activity of the essential oil *P. harmala* from Algeria, Egypt, Libya, Morocco and Tunisia against radicle elongation of *R. sativus*, *L. sativum* and *R. graveolens*, 120 h after sowing. Data are expressed in cm. Results are expressed as the mean of three experiments ± standard deviation.

Radicle Elongation					
*R. sativus*	Algeria	Egypt	Libya	Morocco	Tunisia
Control	4.7 ± 2.8	4.7 ± 2.8	4.7 ± 2.8	4.7 ± 2.8	4.7 ± 2.8
100 μg/mL	2.1 ± 0.7	3.8 ± 3.1	3.6 ± 2.4	2.4 ± 1.3	3.1 ± 2.1
50 μg/mL	4.1 ± 1.1	3.0 ± 2.6	2.0 ± 1.7	3.8 ± 2.8	3.5 ± 2.2
25 μg/mL	4.1 ± 2.1	4.1 ± 2.8	5.2 ± 3.4	4.5 ± 2.1	4.0 ± 2.1
12.5 μg/mL	2.2 ± 0.9	4.3 ± 1.8	4.1 ± 3.4	4.1 ± 3.9	3.3 ± 2.3
*L. sativum*					
Control	5.9 ± 3.8	5.9 ± 3.8	5.9 ± 3.8	NT	5.9 ± 3.8
100 μg/mL	1.6 ± 1.7	4.6 ± 4.2	4.5 ± 3.5	NT	4.2 ± 1.1
50 μg/mL	1.8 ± 2.0	3.2 ± 0.9	2.6 ± 1.1	NT	2.5 ± 1.5
25 μg/mL	2.6 ± 3.6	4.5 ± 1.3	0.9 ± 1.3	NT	2.5 ± 3.6
12.5 μg/mL	3.4 ± 0.7	4.3 ± 2.3	1.7 ± 2.0	NT	1.6 ± 1.6
*R. graveolens*					
Control	1.3 ± 0.7	1.3 ± 0.7	1.3 ± 0.7	NT	1.3 ± 0.7
100 μg/mL	0.5 ± 0.2 ****	0.4 ± 0 ****	0.4 ± 0.3 ****	NT	0.5 ± 0.3 ****
50 μg/mL	0.4 ± 0.2 ****	0.5 ± 0.2 ****	0.6 ± 0.3 ****	NT	0.5 ± 0.2 ****
25 μg/mL	0.7 ± 0.1 ****	0	0.5 ± 0.2 ****	NT	0.4 ± 0.3 ****
12.5 μg/mL	0.5 ± 0.3 ****	0	0.6 ± 0.3 ****	NT	0.8 ± 0.3 ****

Note: **** *p* < 0.0001 vs. control; NT (not tested).

**Table 5:** Harvest site characteristics.

Locality	Bioclimatic Stage	Altitude (m)	Mean Temperature (°C)
Tunisia-Kasserine-Bouzguem	Semi-arid	400	17.5
Algeria-Bousaada	Semi-arid and dry cold	461	19.7
Libya-El Hisha	Humid	376	20
Morocco-Marrakech-Haouz	Arid sub-humid	600	18
Egypt-Marsa Mattrouh	Semi-desert	410	28
